# Home-field advantage? evidence of local adaptation among plants, soil, and arbuscular mycorrhizal fungi through meta-analysis

**DOI:** 10.1186/s12862-016-0698-9

**Published:** 2016-06-10

**Authors:** Megan A. Rúa, Anita Antoninka, Pedro M. Antunes, V. Bala Chaudhary, Catherine Gehring, Louis J. Lamit, Bridget J. Piculell, James D. Bever, Cathy Zabinski, James F. Meadow, Marc J. Lajeunesse, Brook G. Milligan, Justine Karst, Jason D. Hoeksema

**Affiliations:** Department of Biology, University of Mississippi, P.O. Box 1848, University, 38677 MS USA; National Institute for Mathematical and Biological Synthesis, University of Tennessee, 1122 Volunteer Blvd, Knoxville, TN 37996-3410 USA; School of Forestry, Northern Arizona University, 200 E. Pine Knoll, Flagstaff, AZ 86011 USA; Department of Biology, Algoma University, 1520 Queen Street East, Sault Ste. Marie, ON P6A 2G4 Canada; Department of Environmental Science and Studies, DePaul University, McGowan South Suite 203, 1110 West Belden Avenue, Chicago, IL 60614 USA; Department of Biological Sciences, Northern Arizona University, 617 S. Beaver Street, Flagstaff, AZ 86011-5640 USA; School of Forest Resources and Environmental Science, Michigan Technological University, 1400 Townsend Dr, Houghton, MI 49931-1295 USA; Department of Ecology and Evolutionary Biology, University of Kansas, Lawrence, KS 66045 USA; Department of Land Resources and Environmental Sciences, Montana State University, 344 Leon Johnson Hall, Bozeman, MT 59717 USA; Institute of Ecology and Evolution, University of Oregon, 335 Pacific Hall, Eugene, OR 97403 USA; Department of Integrative Biology, University of South Florida, 4202 East Fowler Avenue, Tampa, FL 33620 USA; Department of Biology, New Mexico State University, Las Cruces, NM 88003 USA; Department of Renewable Resources, University of Alberta, 442 Earth Sciences Building, Edmonton, AB T6G 2E3 Canada

**Keywords:** Arbuscular mycorrhizal fungi, Community ecology, Evolution, Geographic origin, Soil micro-organisms, Local adaptation, Symbiosis

## Abstract

**Background:**

Local adaptation, the differential success of genotypes in their native versus foreign environment, arises from various evolutionary processes, but the importance of concurrent abiotic and biotic factors as drivers of local adaptation has only recently been investigated. Local adaptation to biotic interactions may be particularly important for plants, as they associate with microbial symbionts that can significantly affect their fitness and may enable rapid evolution. The arbuscular mycorrhizal (AM) symbiosis is ideal for investigations of local adaptation because it is globally widespread among most plant taxa and can significantly affect plant growth and fitness. Using meta-analysis on 1170 studies (from 139 papers), we investigated the potential for local adaptation to shape plant growth responses to arbuscular mycorrhizal inoculation.

**Results:**

The magnitude and direction for mean effect size of mycorrhizal inoculation on host biomass depended on the geographic origin of the soil and symbiotic partners. Sympatric combinations of plants, AM fungi, and soil yielded large increases in host biomass compared to when all three components were allopatric. The origin of either the fungi or the plant relative to the soil was important for explaining the effect of AM inoculation on plant biomass. If plant and soil were sympatric but allopatric to the fungus, the positive effect of AM inoculation was much greater than when all three components were allopatric, suggesting potential local adaptation of the plant to the soil; however, if fungus and soil were sympatric (but allopatric to the plant) the effect of AM inoculation was indistinct from that of any allopatric combinations, indicating maladaptation of the fungus to the soil.

**Conclusions:**

This study underscores the potential to detect local adaptation for mycorrhizal relationships across a broad swath of the literature. Geographic origin of plants relative to the origin of AM fungal communities and soil is important for describing the effect of mycorrhizal inoculation on plant biomass, suggesting that local adaptation represents a powerful factor for the establishment of novel combinations of fungi, plants, and soils. These results highlight the need for subsequent investigations of local adaptation in the mycorrhizal symbiosis and emphasize the importance of routinely considering the origin of plant, soil, and fungal components.

**Electronic supplementary material:**

The online version of this article (doi:10.1186/s12862-016-0698-9) contains supplementary material, which is available to authorized users.

## Background

Local adaptation, the differential success of genotypes in their native versus foreign environments, is one mechanism that favors population differentiation and may lead to eventual speciation [1, 2]. Adaptation of populations to local abiotic factors is well established [2]. However, biotic factors can also exert strong selective pressures and may greatly alter an organism’s fitness (the ability of individuals to contribute descendants to subsequent generations) in its local environment, leading to coevolution [2–5]. Although the mechanisms that promote (e.g., strong local selective pressure) or suppress (e.g., high gene flow or reduced genetic variation) local adaptation may vary spatially and temporally [2], detecting patterns of local adaptation can provide insight into the evolutionary dynamics of species and their interactions. Such processes may be particularly important for plants, which commonly associate with a wide variety of microbial symbionts that can have profound effects on plant fitness and their evolution [6].

Symbionts are likely to influence plant adaptation because they have strong effects on multiple dimensions of plant resource acquisition and defense, both of which could mediate plant adaptation to their environment. Moreover, symbionts tend to have large population sizes and rapid turnover and therefore are likely to evolve more quickly than their host [7]. This is particularly true for mycorrhizal fungi, whose non-specific horizontal transmission increases the genetic variation of symbionts colonizing host roots [8]. Given that the fitness of host and symbionts can be independent, there is potential for local symbionts to enhance or decrease plant local adaptation. To tease apart these dynamics, investigations regarding local adaptation and mycorrhizal interactions generally utilize experimental designs with reciprocal crosses in which the fitness of the host and/or the symbiont’s genotypes is compared between sympatric and allopatric combinations. Such experiments examing local adaptation of symbionts to their hosts have ranged in outcome from supporting local adaptation [9, 10] to maladaptation [11] to failure to find adaptive responses [12] and even multiple outcomes within the same study [13]. One way to synthesize such variation and test broad general predictions regarding local adaptation is via meta-analysis, which assesses general predictions by integrating results across studies. Here, we utilize meta-analysis to explore potential local adaptation of plants to selective pressures imposed by arbuscular mycorrhizal (AM) fungi, one of the most commonly-studied types of mycorrhizal fungi, and their soil environment. More specifically, we examine whether the geographic origin of the plant host, their AM fungal partner (s), and soil alters plant biomass response to AM inoculation.

AM fungi (from the monophyletic phylum Glomeromycota), along with fungi from the phylum Mucoromycotina, are thought to have evolved with the original land plants 460 mya [14–19]. Although AM fungi are often beneficial to their host plants, these relationships vary along a continuum from mutualistic to parasitic [20, 21]; however, in spite of recent advances [11–18], the factors that determine such variability are not fully understood [22, 23]. Futhermore, most studies examining mycorrhizal ecology neglect the role of local adaptation in shaping the symbiotic outcomes, potentially limiting our understanding of plant-fungal ecology [22].

AM fungi potentially act as an important selective force for plants because they occur over broad geographic scales and across diverse biotic and abiotic gradients. These well studied fungi are obligate biotrophs and associate with over 80 % of terrestrial plants [24]. They facilitate the exchange of mineral nutrients from the soil for fixed carbon from the host, thereby providing novel nutritional pathways for both partners that fundamentally alter phenotypes and influence fitness [6]. Given the importance of mycorrhizal fungi to soil resource uptake and geographic variation in soil characteristics, benefits of mycorrhizal symbioses may be expected to show evidence of local adaptation. The exchange of nutrients is an important avenue by which plants and mycorrhizal fungi are able to act as reciprocal selective forces on one another [25]. Both partners can vary in their effectiveness as a mutualist. Some exchanges are symmetrical and both partners receive equal and clear benefits; others are asymmetrical where one partner recieves a greater benefit than the other (i.e., the presence of “cheaters”). This asymmetry in nutrient exchange potentially destabilizes mycorrhizal interactions because selection favors individuals that provide reduced benefit and incur less cost [26]; however, interactions may be stabilized if the most beneficial partners exhibit preferential allocation to each other [27]. Plant-mediated sanctions, which hinder the growth of mycorrhizal cheaters, have been demonstrated both empirically [28–30] and theoretically [25, 31–33]. Consequently preferential allocation generates scenarios such that plant hosts are selecting for better mycorrhizal fungal mutualists and mycorrhizal fungal mutualists are selecting for better hosts, but the degree and nature of such selection is an area of developing research.

Local adaptation may be an important process underlying variation in plant response to mycorrhizal inoculation, and soils may mediate this response. For example, when plants and mycorrhizal fungi are involved in long-term relationships, mutual cooperation is more likely to evolve and the response of plants to AM inoculation may be greater than for novel combinations of plants and fungi, which do not share the same evolutionary history [34]. Plant and fungal adaptation to each other, plant adaptation to soil, and fungal adaptation to soil can all influence plant response to AM fungi; therefore, in order to fully understand patterns of adaptation within mycorrhizal relationships, it is important to understand such interactions individually.

### Mediation by soil of plant adaptation to arbuscular mycorrhizal fungi

Previous research emphasizes the direct effect that soil properties can have as selective agents on plant populations, whereby the fitness of individuals within populations increases when they can better tolerate specific soil conditions [35–37]; however, soil properties may also indirectly influence plant fitness through its effects on mycorrhizal interactions. Here, we consider the response of plants to AM inoculation where a) soils are of the same origin as the plant, and/or b) soils are of the same origin as the fungus. Local soil type and fertility are likely to play a key role in plant adaptation to mycorrhizal fungi given the central role of mycorrhizal symbioses in plant nutrient uptake [31, 32, 38–40]. Thus, the acquisition of water and nutrients from soils can act as a strong selection pressure, such that plants and their associated mycorrhizal fungi exert reciprocal selective forces on each other, leading to local populations defined by their ability to coexist under existing soil conditions [13, 41]. For example, two ecotypes of big bluestem (*Andropogon gerardi*) each grew comparatively better in its own soils, but the response to AM fungi was mediated by soil nutrient levels [42]. Overall, both ecotypes benefited more from AM fungi when grown in soil from the low-nutrient site versus the higher nutrient site, but ecotypes originating from low fertility soils were more responsive to AM fungi than those ecotypes from high fertility soils [42]. This pattern would be expected from plants allocating more resources to mycorrhizal fungi when soil resources are scarce and more resources to direct resource uptake via roots when soil resources are plentiful. Consequently, plant response to mycorrhizal inoculation may vary not only in response to present soil conditions, but also in response to the soil conditions in which plant populations have evolved, though the generality of this pattern has not been tested.

To determine the degree to which local soils influence the response of plants to mycorrhizal inoculation, we compared the effect of mycorrhizal inoculation on plant biomass for instances when the plant origin matched that of the soil (sympatric) to instances when the plant origin was different from that of the soil (allopatric). If the local soil has an important evolutionary influence on plant response to mycorrhizal fungi, we would expect to see significant differences in plant response to mycorrhizal inoculation for sympatric combinations of the plant and soil compared to allopatric combinations. For example, if a plant population has evolved in soil conditions that promote dependence on mycorrhizal fungi (such as low nutrient conditions), we would expect individuals from that population to have developed a higher dependence on their mycorrhizal fungi such that sympatric combinations stimulate plant biomass more than allopatric combinations; however, we may also find maladaptation such that when combinations are sympatric, plant response to mycorrhizal inoculation is not different from or is lower than when combinations are allopatric.

### Plant response to sympatric combinations of mycorrhizal fungi with their soils

Recent research demonstrates that AM fungi exhibit a low degree of endemism such that the same taxa are often identified on multiple continents, but local environmental conditions are important for determing variation among sites in the abundance and activity of AM fungal communities [43]. This conclusion is supported by transplant studies indicating that AM fungal communities function best in their native soils [9, 12, 13]. For example, when AM fungal inoculum is used to inoculate a foreign soil, the taxonomic composition of the resultant spore communities is altered, demonstrating the importance of environmental filters on fungal community composition [44]. More complex manipulations inoculating a single host grown in soils from different geographic locations revealed that the same fungal inoculum can generate three different communities in soils that vary in texture, pH, and nutrient levels, suggesting that a particular AM fungal community may be better matched to its soil environment than communities taken from other locations [45]. This divergence in fungal community composition may reflect interspecific variation in tolerance to the novel environment, increasing the possibility for local adaptation between the fungi and soil.

Fungal adaptation to local soils may be a function of soil resources. If so, we might expect that local fungi would better help the plant overcome the resource deficiencies of the local soil. This type of local adaptation would be predicted by biological market theory [31, 32] and resource stoichiometry [38], as well as by fungal dynamics generated via context dependency of plant preferential allocation [27]. For example, in phosphorus deficient soils, fungi that can efficiently acquire and deliver phosphorus may be favored by plants, while nitrogen deficient soils may favor fungi with different resource gathering specializations. Adaptation of AM fungi to the soils in which they best promote plant growth has been observed in tall grass prairie systems [13, 44]. Mycorrhizal fungi may also alter host plant fitness by changing soil properties including soil aggregation and structure via changes in soil organic matter, minerals, and root exudates, leading to significant changes in plant fitness [46, 47]. Consequently, understanding patterns of local adaptation can provide practical information for aiding species and habitat management decisions, such as whether inoculation of plants with local genotypes of mutualistic symbionts like mycorrhizal fungi is beneficial to enhance ecosystem services (e.g., food production, restoration of compromised ecosystems, bioremediation, carbon sequestration) [13, 44]. To test the degree to which plants benefit from local adaptation of fungi to soils, we compared plant mycorrhizal response when fungi and soils were of the same geographic origin (sympatric) compared to when they were of different origins (allopatric).

### Local adapation (or maladaptation) of plants to arbuscular mycorrhizal fungi

The response of plants to mycorrhizal fungi is known to vary across plant-fungal combinations [34], but the extent to which plants generally grow better or worse with their local AM fungi is not known. The intimate nature of the interaction between plants and their AM fungi presents the possibility for both adaptation and maladaptation. Given that different species (and genotypes) of AM fungi have different levels of fitness when grown with different plant species (and genotypes) [41], the likelihood of greater plant response to mycorrhizal inoculation with local fungi will depend upon the correlation of plant and fungal relative fitness [48]. When the relative fitness of the plant and fungus are positively correlated, positive feedbacks will lead to co-adaption. This feedback is expected to occur through co-evolutionary time, thereby leading to local adaptation. In contrast, when plant and fungal relative fitness are negatively correlated, negative feedbacks will lead to maladaptation [39, 48]. Thus, plants may perform worse with fungi or soil from their home location because their mycorrhizal fungi have evolved to take greater advantage of their interactions with plants. This dynamic can be similar to that observed in host-enemy coevolution where rapidly evolving enemies often exert more negative effects on hosts when enemies originate from the same as opposed to different regions [49–51].

Empirical tests of feedback dynamics in plants provide evidence of both positive [52] and negative feedbacks [26, 53]. Negative feedbacks between AM fungi and their host have been demonstrated experimentally when plant hosts have responded more positively to AM fungal communities collected from non-related host plants than to their own AM fungal communities, largely as a result of asymmetries in the benefits exchanged [26, 53]. Thus we hypothesize that variation in the geographic origin of soils, mycorrhizal fungi, and host plants will result in plant responses to mycorrhizal inoculation that vary along a continuum from mutualistic to parasitic [20, 21], as a result of local adaptation or maladaptation of mycorrhizal fungi and host plants to each other and their soils. To determine the degree to which origin of AM fungi influence the response of plants to mycorrhizal inoculation, we compared the effect of mycorrhizal inoculation on plant biomass for instances when the plant origin matched that of the fungi (sympatric) to instances when the plant origin was different from that of the fungi (allopatric). If adaptation to local fungi has an important evolutionary influence on plant fitness, we would expect to see significant differences in plant response when paired with sympatric combinations fungi, as compared to allopatric combinations.

While interactions between host plants, AM fungi, and soils may result in local adaptation or maladaptation of plant populations, we do not currently have the predictive capacity to determine how often and under what circumstances adaptation or maladaptation will occur. In an attempt to fill in this knowledge gap, we explored patterns of adaptation in AM relationships by using meta-analysis to detect variation in plant response to AM inoculation across a variety of systems and environmental conditions involving plants inoculated with AM fungi. Previous meta-analyses have examined local adaptation across a broad range of taxa including plants, animals, and fungi [54, 55], but have rarely included mycorrhizal symbioses, despite their widespread occurrence [49]. A recent meta-analysis revealed that variation in plant biomass due to AM fungal inoculation is influenced by multiple simultaneous factors, including N and P fertilization, inoculum complexity, and host plant functional group [23], but the general importance of local adaptation in response of plants to AM fungi is largely unknown. With a dataset composed of 1170 studies (from 139 papers), we tested whether variation among studies in the relative geographic origin of the plant, mycorrhizal inoculum, and/or soil was important in altering plant biomass response to AM fungi. To quantify the effect of local adaptation in altering plant response to AM inoculation, we utilized plant growth response as a fitness proxy for the plant. Plant growth is a common metric used to assess fitness in mycorrhizal relationships as it directly concerns the vegetative vigor of the plant [56]. Additionally, we estimated variation in local adaptation of plants to AM fungi and their soils across a smaller subset of the studies that varied the geographic origin of the plant, AM inoculum, and/or soil within the same published paper (referred to here as within-paper analyses), which represents a more direct test of potential local adaptation since a metric of local adaptation can be calculated for each individual study. We primarily aimed to address whether, and to what degree, plant response to AM fungi may be influenced by local adaptation between plants, fungi, and soil.

## Results

**Fig. 1:**
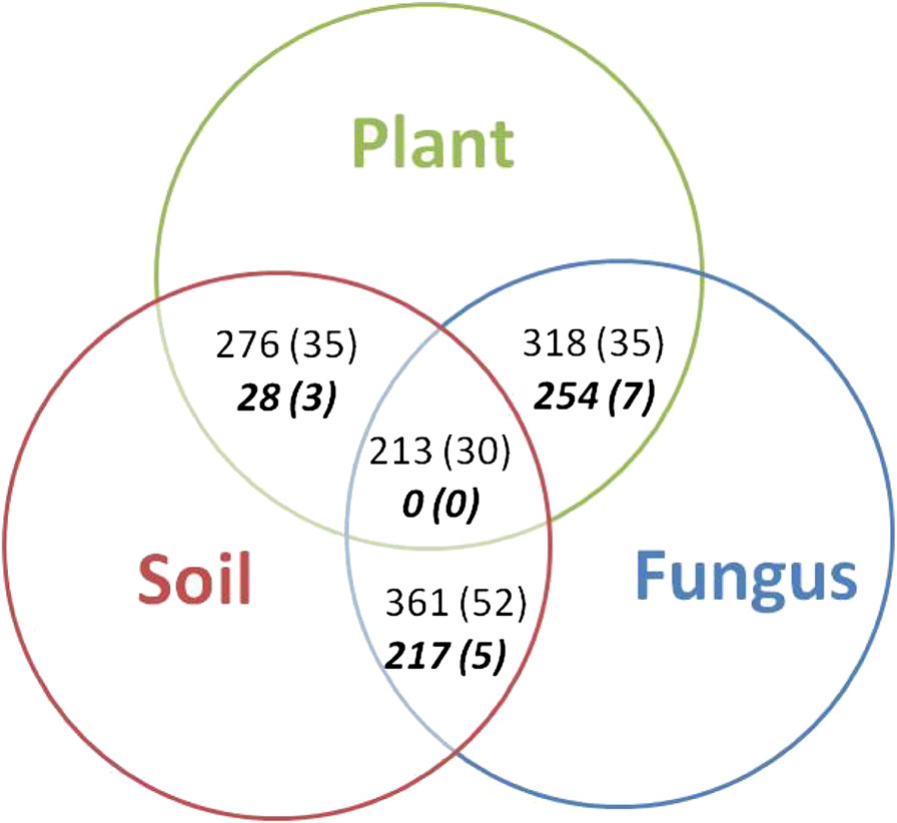
Available studies for all components of local adaptation. Number of studies (and associated papers) in which origin is reported for pairwise investigations of local adaptation as well as all three components of local adaptation. The number of studies (and associated papers) in which allopatric and sympatric pairings are in the same paper are in bold

Our objective was to explore the potential role of local adaptation in shaping AM fungal relationships with their plant hosts (Table 1, Additional file 1: Table S2). Here we report results for both between-study analyses of studies in which the plant, fungal partner, and soil origin were all known (Analysis Group 1: Between-study Analyses; Fig. 1), and results from analyses of a smaller subset of the studies that varied the geographic origin of the plant, AM fungal inoculum, and/or soil within the same published papers (Analysis Group 2: Within-paper Analyses; Fig. 1). For Analysis Group 1, analyses were conducted for all available studies (‘Full Dataset’) and for two subsets, one in which only a single fungal species was used as inoculum (‘Single Species’) and one in which studies were only conducted in the laboratory (‘Lab Studies’: greenhouses, growth chambers, lathe houses, or shade houses).

**Table 1:** The eight categorical predictor variables that were explored in our random-effects meta-analyses

Name	Levels	
Origin*	5	‘Sympatric’ (all components are from the same geographic location), ‘Allopatric’ (all components are from different geographic locations), ‘Fungi-Soil Sympatric’ (fungus and soil sympatric but allopatric to the plant), Plant-Fungi Sympatric (plant and fungus sympatric but allopatric to the soil), Plant-Soil Sympatric (plant and soil sympatric but allopatric to the fungus)
Plant Functional Group	6	C_4_ grasses, C_3_ grasses, N-fixing forbs, non-N-fixing forbs, N-fixing woody plants, non-N-fixing woody plants
Inoculum Complexity	3	Whole soil inoculum, multiple species inoculum, single species inoculum
Sterility	2	Sterilized (background soil was sterilized prior to the experiment), not sterilized
Microbe Control	3	Microbial wash (application of aqueous filtrate of non-mycorrhizal microbes), other microbial addition (non-mycorrhizal microbes added via other avenues such as rhizosphere soil from non-mycorrhizal culture plants), no added non-mycorrhizal microbes
Experimental set-up:	2	Laboratory (greenhouse, growth chamber, lathe house or shade house), field
N-fertilization, P-fertilization	2	Fertilized or not

Seven variables were used previously in Hoeksema et al. [23] and one variable was unique to our analyses (as indicated by asterisk)

For Analysis Group 1, the effect size compared plant growth with versus without mycorrhizal inoculation. Local adaptation in this set of analyses was assessed by comparing this effect size metric among studies differing in whether plants, fungi, and/or soils were sympatric or allopatric in origin. For Analysis Group 2, the effect size compared plant response to mycorrhizal inoculation in sympatric versus allopatric pairings, and thus was a direct assessment of local adaptation or maladaptation. For Analysis Group 1, we used the five-level variable ORIGIN to assess the degree and nature of local adaptation for plant-mycorrhizal relationships. Specifically, this allowed us to investigate whether ‘Sympatric’ (all components are from the same geographic location), ‘Allopatric’ (all components are from different geographic locations), ‘Fungi-Soil Sympatric’ (fungus and soil sympatric but allopatric to the plant), ‘Plant-Fungi Sympatric’ (plant and fungus sympatric but allopatric to the soil), and ‘Plant-Soil Sympatric’ (plant and soil sympatric but allopatric to the fungus) forms of the symbiosis differ in plant response to mycorrhizal inoculation. A significant effect of ORIGIN was not enough to determine whether local adaptation was responsible for altering plant response to mycorrhizal inoculation; rather, effect sizes greater than all allopatric combinations indicate some degree of local adaptation while effect sizes smaller than allopatric combinations indicate some degree of maladaptation. Effect sizes that do not differ from all allopatric combinations indicate a lack of signal for any adaptive relationship.

### Analysis Group 1: Between-study analyses

Regardless of whether the plant, fungal partner, and/or soil originated in sympatry or allopatry, the mean effect size of AM inoculum on host biomass was consistently greater than zero (mean estimate ± standard error) for the Full Dataset (0.537 ± 0.219, k = 213), Single Species (0.488 ± 0.463, k = 115), and Lab Studies (0.427 ± 0.375, k = 182). The overall effect was largest in the Full Dataset but was also slightly positive in the Single Species and Lab Studies analyses (Additional file 2: Figure S1).

**Fig. 2:**
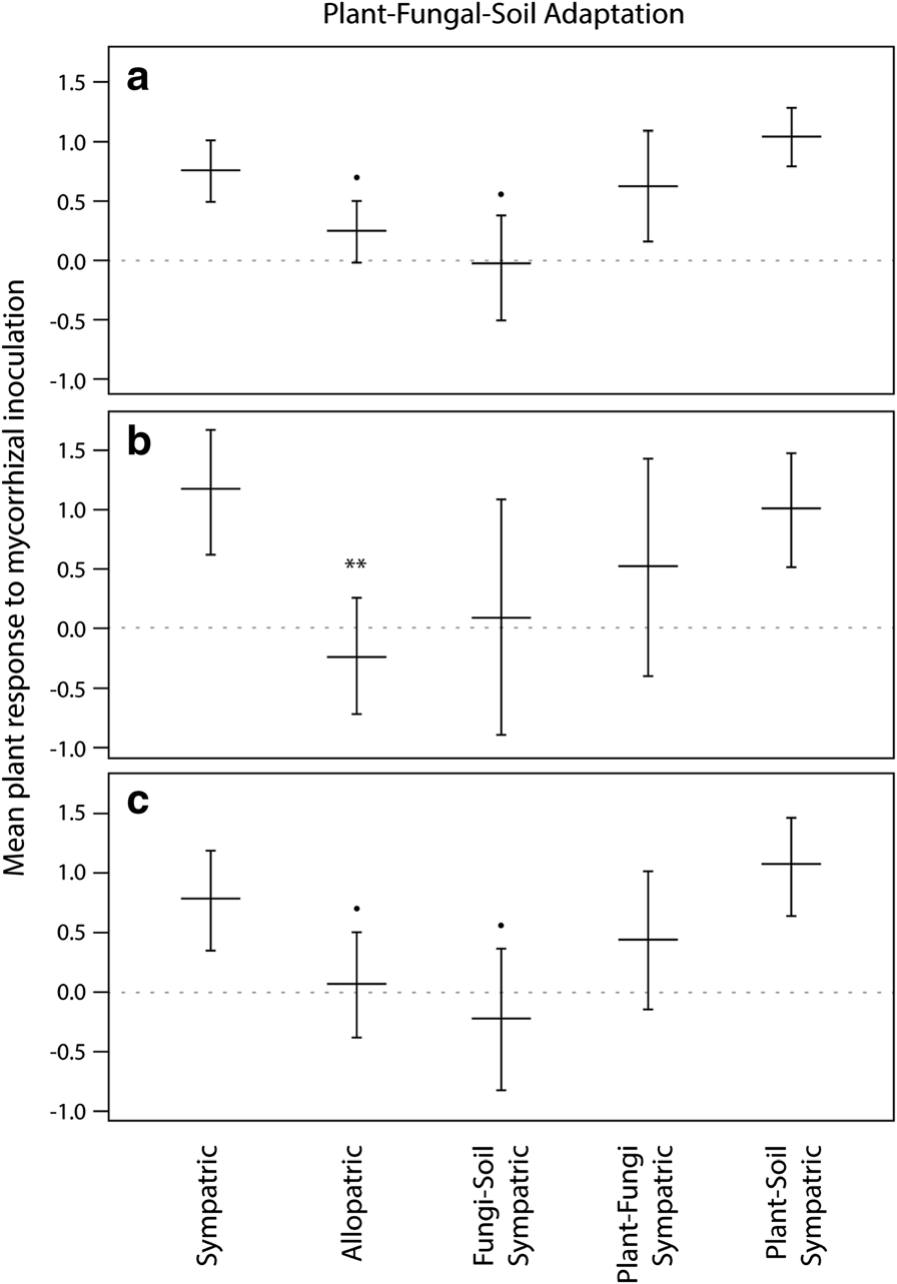
Plant-Fungal-Soil Adaptation. When the plant, fungal inocula, and soil were sympatric, the change in plant biomass due to inoculation with mycorrhizal fungi tended to be greater than when all three were allopatric. Values shown are weighted mean effect sizes ± standard error for arbuscular mycorrhizal fungi from the Full Dataset (**a**) Single Species Inocula (**b**) and Lab Studies (**c**). The dotted line indicates no response, values above the line indicate positive response to mycorrhizal inoculation (mutualism), and values below the line indicate negative response to mycorrhizal inoculation (parasitism). Symbols indicate differences from sympatric combinations of the plant, soil, and fungal inocula based on planned contrasts. Significant codes: 0 ‘***’ 0.001 ‘**’ 0.01 ‘*’ 0.05 ‘·’ 0.1

The first question we addressed was whether sympatric versus allopatric origins of the plant, fungal partner and soil affected host plant response to the AM symbiosis. ORIGIN was significant for explaining plant biomass response to AM inoculation for all three datasets: the Full Dataset (Q_M_ (df_4_) = 11.6, p = 0.021), Single Species (Q_M_ (df_4_) = 10.1, p = 0.039), and Lab Studies (Q_M_ (df_4_) = 9.38, p = 0.052, Table 2). The results for this grouping variable revealed three patterns which were consistent across all three datasets. First, the effect of AM inoculation on plant biomass was higher when the plant, AM fungal partner, and soil were all sympatric compared to when they were all allopatric (Fig. 2), indicating local adaptation of the plant and fungi to one another and the soil. Second, when the plant and fungal partner were from the same origin, but allopatric to the soil, there was no difference in the effect of AM inoculation as the effect size did not differ from all allopatric or all sympatric combinations, suggesting no adapative relationship. Finally, the relationship of either the fungi or the plant to the soil was important for explaining the effect of AM inoculation on plant biomass (Fig. 2). Specifically, if the plant and soil were from the same origin (but allopatric to the fungal partner) the effect of AM inoculation was much greater than when all three components were allopatric, indicating potential local adaptation of the plant to the soil; however, if AM fungi and soil were from the same origin (but allopatric to the plant), the effect of AM inoculation was not different from the effect of allopatric combinations of the plant, soil, and fungal partner, indicating a lack of adaptation of the fungi to the soil (Fig. 2). Furthermore, the effect of inoculation on plant biomass did not differ when 1) the plant, AM fungal partner, and soil were all from the same origin (sympatric), 2) only the plant and soil were from the same origin (and allopatric to the fungus), and 3) only the fungal partner and soil were sympatric (allopatric to the plant; Fig. 2).

**Table 2:** Test statistics for categorical effects in models for each dataset

	Full dataset			Single species inocula			Lab studies only		
	Q_E_(df_198_) = 952.7 *p* < 0.0001			Q_E_(df_102_) = 519 *p* < 0.0001			Q_E_(df_166_) = 897.7 *p* < 0.0001		
	Q_M_	df	*p* value	Q_M_	df	*p* value	Q_M_	df	*p* value
Plant Functional Group	3.69	5	0.595	4.25	5	0.515	3.347	5	0.647
Inoculum Complexity	**7.11**	**2**	**0.029**	–	–	–	*5.347*	*2*	*0.069*
Sterility	–	–	–	–	–	–	0.510	1	0.475
Microbe Control	–	–	–	–	–	–	0.551	1	0.458
Experimental Set-Up	0.842	1	0.359	0.251	1	0.627	–	–	–
P Fertilization	0.036	1	0.849	2.04	1	0.154	0.011	1	0.916
N Fertilization	0.007	1	0.936	2.26	1	0.132	0.115	1	0.734
Plant-Fungal-Soil Origin	**11.6**	**4**	**0.021**	**10.1**	**4**	**0.039**	**9.38**	**4**	**0.052**

Values are obtained from models for between-study Analyses (Analysis Group 1) for all datasets including: Full Dataset, Single Species Inocula, and Lab Studies Only). Q statistics are approximately *χ* ^2^ distributed with degrees of freedom (df). Dashed lines indicated explanatory variables which were not included in that analysis group. Bold values represent *p* values < 0.05 and italicized values represent *p* values > 0.05 and < 0.1Q_E_: test statistic for the test of residual heterogeniety

Q_M_: test statistic for the omnibus test of coefficients

Finally, for analyses when multiple species of fungi were used as inocula, INOCULUM COMPLEXITY (single species, multiple species, or whole soil inoculum) was significant for explaining plant biomass response to AM inoculation for the Full Dataset (Q_M_ (df_2_) = 7.11, p = 0.027) and tended to be important for Lab Studies (Q_M_ (df_2_) = 5.35, p = 0.069, Table 2). Specifically, the effect of AM inoculation was greater when the AM inocula contained multiple species than when it contained a single species or the whole soil (Additional file 3: Figure S2).

### Analysis Group 2: Within-Paper Analyses

Our final analyses examined direct studies of putative local adaptation where sympatric and allopatric origin of the plant, AM fungi and/or soil were manipulated within the same study.

**Fig. 3:**
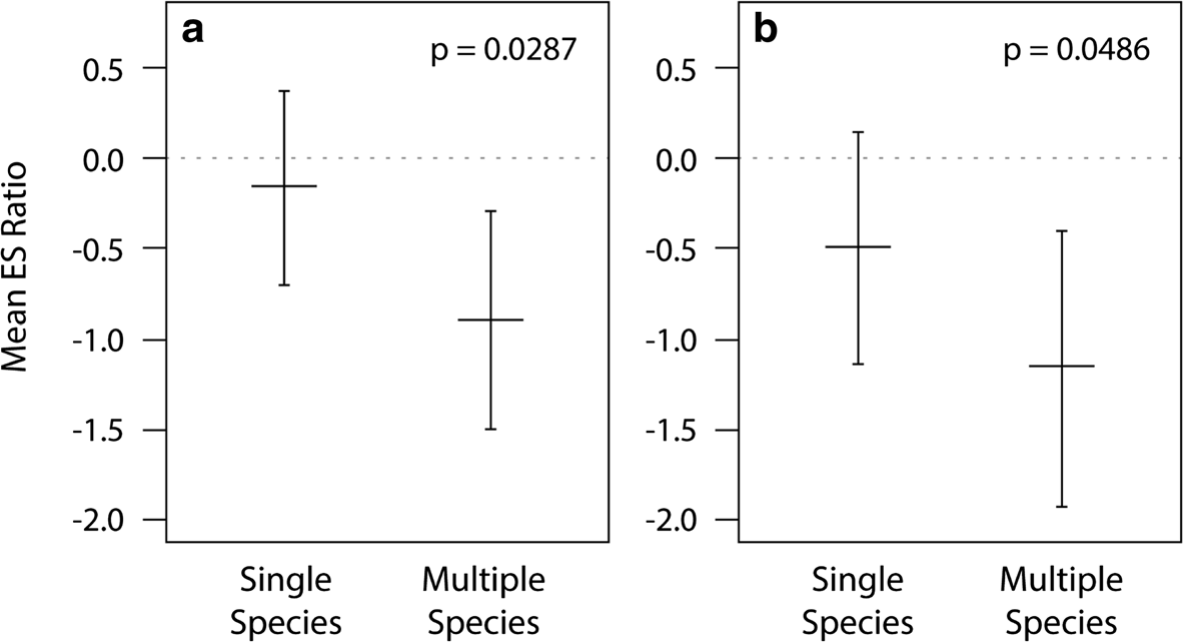
Inoculation Complexity for Within Paper Analyses: When a single species of fungi was used as inocula, the effect of sympatry was greater than allopatry (although not different than zero) while the reverse was true when the fungal inocula contained multiple species. Values shown represent the ratio of weighted mean effect sizes (ES) ± standard error for arbuscular mycorrhiza from within paper examinations of Plant and Fungi (**a**) and Fungi and Soil (**b**). The dotted line indicates no response, values above the line indicate positive local adaptation, and values below the line indicate maladaptation. *P* values indicate differences based on planned contrasts

*Plant-Fungal:* We were able to calculate within-paper effect sizes of plant-fungal local adaptation for 254 laboratory studies (from 7 papers) of AM fungi with sterilized background soil (Fig. 1). While the overall estimated effect size for this model was negative, it did not significantly differ from zero (mean estimate ± standard error: −0.534 ± 0.550, k = 254), indicating no average difference in the effect of AM inoculation on plant biomass when the plant and fungal partner originated in sympatry compared to when they originated in allopatry, and thus no significant overall effect of local adaptation or maladaptation. For this analysis, INOCULUM COMPLEXITY was the only significant predictor of plant local adaptation to AM fungi (Q_M_(df_1_) = 4.78, p value = 0.029, Table 3), with allopatric combinations outperforming sympatric combinations for multiple species inocula and no difference between sympatric and allopatric combinations for single species inocula (Fig. 3a).

**Table 3:** Within paper analysis test statistics for categorical effects

	Plant-fungal			Fungal-soil			Plant-soil		
	Q_E_(df_247_) = 1238.4, *p* < 0.0001			Q_E_(df_210_) = 1229.5, *p* <0 .0001			Q_E_(df_26_) = 27.6, *p* = 0.379		
	Q_M_	df	*p* value	Q_M_	df	*p* value	Q_M_	df	*p* value
Plant Functional Group	2.36	3	0.501	2.09	2	0.352	0.712	1	0.399
Inoculum Complexity	**4.78**	**1**	**0.029**	**3.89**	**1**	**0.049**	–	–	–
Microbe Control	0.031	1	0.860	0.544	1	0.461	–	–	–
*P* Fertilization	0.053	1	0.818	0.798	1	0.372	–	–	–
*N* Fertilization	–	–	–	0.743	1	0.389	–	–	–

Models represent the Analysis Group 2: Between Study analyses. Q statistics are approximately *χ* ^2^ distributed with degrees of freedom (df). Dashed lines indicated explanatory variables which were not included in that analysis group. Bold values represent *p* values < 0.05 and italicized values represent *p* values > 0.05 and < 0.1Q_E_: test statistic for the test of residual heterogeniety

Q_M_: test statistic for the omnibus test of coefficients

*Fungal-Soil:* We were able to calculate within-paper effect sizes of potential fungal-soil local adaptation for 217 laboratory studies (from 5 papers) of AM fungi with sterilized background soil. The overall estimated effect size for this model was negative, but not different from zero (mean estimate ± standard error: −0.820 ± 0.738, k = 217), indicating no overall significant of local adaptation or maladaptation. Similar to plant-fungal adaptation, INOCULUM COMPLEXITY was the only significant predictor of local adaptation (Q_M_(df_1_) = 3.89, p = 0.049, Table 3, Fig. 3), with allopatric combinations of the fungus and soil outperforming sympatric combinations for multiple species inocula and no difference between sympatric and allopatric combinations for single species inocula (Fig. 3b).

*Plant-Soil:* We were able to calculate within-paper effect sizes of plant-soil local adaptation for 28 laboratory studies (from 3 papers) of AM fungi with sterilized background soil; however, our model was severely limited by the available data. Consequently, the data available for this analysis were relatively homogenous and the variability in the model was larger than expected based on sampling variability alone (Q_E_(df_26_) = 27.6, p = 0.379, Table 3). While the overall estimated effect size for this model was positive, it did not significantly differ from zero (mean estimate ± standard error: 0.1189 ± 0.327, k = 28), indicating no overall local adaptation or maladaptation.

*Plant-Fungal-Soil:* No papers in our dataset had both allopatric and sympatric pairings of plant-fungal-soil combinations.

## Discussion

Previous research has emphasized the role of abiotic factors in driving local adaptation of organisms to their local environment, but biotic factors may also greatly alter an organism’s fitness in their local environment [2–5]. Moreover, in a symbiotic interaction, particularly in the case of an obligate symbiosis, understanding co-adaptation between symbionts and between them and the local environments is essential for local adaptation. Although limited by the amount of available data, our results represent an important first step in addressing local adaptation of a symbiosis. Specifically, our results highlight the complexity of the patterns and processes behind local adaptation of plants to mycorrhizal fungi, suggesting that studying plant responses to AM inoculation without considering the geographic origin of the symbionts, plant and soil is neglecting key elements of the interaction.

**Fig. 4:**
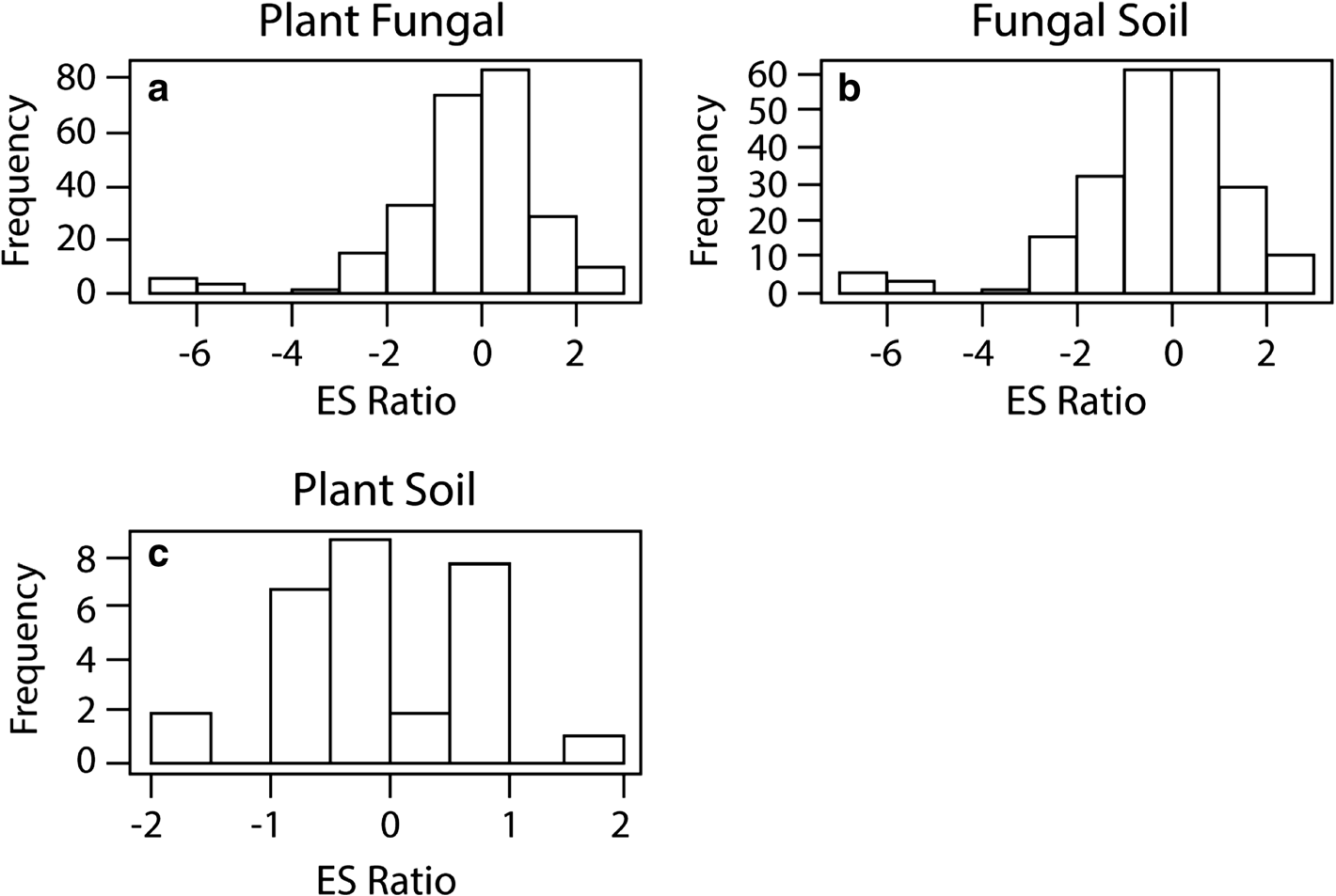
Frequency of AM Fungal Effect Sizes for Within Paper Analyses: The frequency of sympatric combinations outperforming allopatric combinations is greater for the plant and fungus as well as the fungus and soil than for combinations of plants and soil. Values shown represent the ratio of weighted mean effect sizes (ES) for arbuscular mycorrhiza from within paper examinations of Plant and Fungi (**a**) Fungi and Soil (**b**) and Plant and Soil (**c**). Values above zero indicate positive local adaptation, and values below zero indicate maladaptation

Evidence for local adaptation in mycorrhizal symbioses in our meta-analysis is best illustrated by analyses of variation among studies for which the origin of all three components of the mutualism were known. Across datasets, geographic origin was always a significant predictor in describing plant biomass response to AM inoculation (Table 1) and sympatric pairings tended to produce more positive host plant responses than allopatric pairings across all datasets (Fig. 2). This pattern was especially evident when considering studies with only single species inoculum (Fig. 2b). Interestingly, in the more direct test of local adaptation, the within-paper analyses, we did not see this result (Figs. 4, Additional file 4: Figure S3), but this may be a consequence of the small number of studies available for those analyses, as average effect sizes in those analyses were in the direction of local adaptation. While the importance of matching fungal, soil, and plant origin for increasing plant response to AM fungi has been demonstrated in a select number of grassland species [13, 34, 42], the results presented here represent a more extensive exploration of this phenomenon and thus have important implications for both the ecology and conservation biology of mycorrhizas.

To more fully understand the patterns and processes that may shape plant and fungal adaptation to one another and their local soils, we also explored pairwise components of the symbiosis so that we could evaluate plant response to mycorrhizal inoculation when only two of the three components of the mutualism (plant, fungal partner, or soil) were in sympatry but in allopatry relative to the third component of the mutualism. These explorations indicated that the relationship of either the fungi or the plant to the soil is important in explaining the effect of AM inoculation on plant biomass (Fig. 2). In our study, when the plant and soil were from the same origin (but allopatric to the fungus) the effect of AM inoculation was much greater than when all three components were allopatric (but not different from situations in which all three components were sympatric), indicating the potential for local adaptation of plants to soils to influence how plants respond to AM fungi; however, if the fungal partner and soil were from the same origin (but allopatric to the plant) or when the plant and fungal partner were from the same origin (but allopatric to the soil) there was no difference in the effect of AM inoculation compared to allopatric combinations of the plant, soil, and fungal partner (Fig. 2). This result suggests that local adaptation in AM relationships may result from their relationship with the soil environment. These results are exemplified in studies exploring the adaptive relationship of the obligate mycotrophic herb *Aster amellus* to its native soils and native AM fungi [10, 11]. In this system, a 5-year reciprocal transplant experiment evaluating the relationship between soil abiotic conditions, AM fungi and plant growth indicated that plants from a region characteristic of low nutrient availability consistently outperformed plants from a region characteristic of high nutrient availability, likely due to their adaptive relationship with AM fungi. While plants from the low nutrient region had higher AM colonization levels in both environments, emphasizing their dependence on mycorrhizas [11], plants from the high nutrient region had lower aboveground biomass with lower AM colonization, suggesting maladaptation of the plant to its local AM fungi [10, 11]. In conjunction with our results, these findings emphasize the importance of soil factors for for understanding these interactions.

These results suggest that further work investigating ecological interactions of AM fungi and their plant hosts should strive to control for variation in fungal, soil, and plant origin as well as inform species and habitat management decisions in order to maximize mycorrhizal applications [13, 44]. Currently, it is the policy of many habitat managers to restore land using local seed stock (because of the presumed importance of local adaptation) but mycorrhizal inoculum associated with these restoration efforts often comes from inoculum producers, creating a mismatch in the relationship between geographic origin of the plant and soil with the fungal inoculum. Our results suggest that this policy may be just as effective as using locally sourced inoculum but more constrained manipulations are needed.

It is important to note that the effect of mycorrhizal inoculation on plant biomass was consistently larger when the plant and soil were sympatric (and allopatric to the fungus; Fig. 2), but also tended to be high when the plant and fungal partner were sympatric (and allopatric to the soil; Fig. 2). These findings deserve further controlled investigations but suggest that for mycorrhizal relationships, local coadaptation by the plant and AM fungi amid their soil environment has occurred in a manner that benefits both symbiotic partners, and that the alignment of only two of those components can generate enough complementarity to alliviate any decrease in plant performance induced by the mismatch of the third component.

If the plant has adapted to its soil environment independent of the fungi, the plant’s response to AM fungi will depend on soil nutrient levels. In higher nutrient soils, fungi are less likely to provide a significant benefit to the host plant. Alternatively, plants adapted to low nutrient soils should benefit from AM inoculation. This conditioning effect may explain why, in our study, there was so much variation surrounding combinations in which AM fungi and soil were sympatric but allopatric to the plant (Fig. 2). Additionally, we would expect this outcome to be represented not only by the change in plant biomass to inoculation but also in terms of fungal response, as measured either by extraradical hyphal length, spore production, or root colonization rate [13]. Such a response has been illustrated in prairie soils where a strong home-soil preference in terms of AM hyphal growth was found for two remnant native prairies but a reconstructed prairie, which had a shorter time for adaptation, produced equal amounts of extraradical hyphae regardless of soil origin [13]. Unfortunately, fungal responses are not generally well reported within studies, limiting their utility for meta-analysis.

While these analyses represent a robust and thorough exploration of the potential importance of local adaptation in shaping mycorrhizal relationships, they could be made even stronger if more papers reported a full suite of information necessary for multifactorial meta-analysis. This study utilized a subset of a larger dataset consisting of 2984 AM fungal studies from 359 papers [57]. Of the full AM dataset, only 39 % (1170 studies from 139 papers) reported the geographic origin of at least two of the three components of interest: AM fungal inoculum, host plant, and soil. We had to further cull our analyses to studies that only examined inoculation with AM fungi because studies with ectomycorrhizal fungi, the other major mycorrhizal group represented in our database, were limited to 489 studies, the majority of which were lab studies with single species inoculum. This impaired our ability to interpret the potential effect of local adaptation in shaping the effect of mycorrhizal inoculation on plant biomass. Consequently, future investigations examining plant responses to mycorrhizal inoculation should report the geographic origin of their fungal inoculum, host plant, and soil. The importance of reporting this information is further emphasized when looking at within paper studies. Of the 1170 studies (from 139 published papers) only 15 were studies that directly manipulated allopatric and sympatric combinations of the plant, fungi, or soil in the same study, and no study directly manipulated allopatric and sympatric combinations of all three components of the mutualism at the same time (Fig. 1). Thus, our ability to interpret our analyses with respect to within paper manipulations was relatively limited.

### Inoculum complexity

Previous research indicated that the biotic context of mycorrhizal inoculation is important, such that more diverse soil communities lead to a greater effect of mycorrhizal inoculation on the plant [23]. This was true across all of our analyses (Table 1, Table 2). Specifically, inoculum complexity greater than a single taxon (e.g., multiple fungal taxa, whole soil inoculum) stimulated the effect of mycorrhizal inoculation on plant biomass compared to when a single fungal taxon was used for inoculation (Additional file 3: Figure S2), but results were more complex for within paper analyses. In those analyses, when a single fungal species was used as inoculum, the effect of sympatry was greater than allopatry, suggesting local adaptation, while the reverse was true when fungal inocula contained multiple species (Fig. 3). These results may stem from complementarity among mycorrhizal fungal species, which can provide a greater benefit for the host [58, 59]. Conversely, a more complex inoculum has the potential to provide a greater benefit to the host across a broader range of environments, possibly as a result of a diversity of foraging strategies which may allow for a greater ability to adapt to a new environment. For example, external hyphal architecture, which appears to be linked to unique functions within each hyphal type, can differ greatly according to fungal species [60]. Such functions may be directly related to differences in the benefit the plant receives from the fungi in a given environment. Consequently, inoculum that contains more fungal species may also provide benefits across a wider range of abiotic conditions.

The fact that diverse inoculum increases plant response to mycorrhizal inoculation (Tables 1 and 2) underscores the importance of multiple AM fungal species for shaping the role of local adaptation in altering plant response to mycorrhizal inoculation (Fig. 3); however, our study is limited in exploring the effects of other soil organisms for altering such relationships. Our analysis includes any study that added inoculum, regardless of whether it was pure culture inoculum or not, indicating that other components in the inoculum such as non-AM fungal microorganisms could be contributing to plant responses to inoculation; however, it is likely that the presence of other AM species (single species vs. multiple species) is having a larger impact in altering plant response to mycorrhizal inoculation and mediating the role of local adaptation in altering this relationship. This conclusion is supported by our results which indicate that neither the addition of supplementary microbes (MICROBE CONTROL), sterilization of the background soil (STERILITY), nor EXPERIMENTAL SET-UP (laboratory vs. field study) significantly altered plant response to mycorrhizal inoculation for any of the analyses. If non-AM fungal microorganisms in the inocula were important drivers in altering plant response to mycorrhizal inoculation, we would expect that at least one of these variables would be significant. Consequently, it is more likely that the presence of other mycorrhizal species and not of other soil micoorganisms is the important driver for the patterns we found here.

## Conclusions

Our meta-analysis represents one of the first broad explorations of the potential role of local adaptation in determining the outcome of mycorrhizal relationships. Our results suggest that when the origin of the plant, AM fungi, and soil is known, local adaptation may be important for describing the effect of mycorrhizal inoculation on host biomass. Specifically, sympatric relationships of the plant, fungus, and soil tend to outperform allopatric relationships. Of the sympatric relationships, when plant and fungi were sympatric (but allopatric to the soil), there is a less positive response by the host plant, suggesting that fungi adapt to the soil environment in such a way as to be less mutualistic to novel host plants. These results have wide ranging implications for future ecological, evolutionary, and applied studies, and suggest that local adaptation of the plant and the fungi to one another and to their soil environment should be considered specifically when designing experiments, preparing restoration campaigns or selecting specific mycorrhizal assemblages for use as inoculants.

## Methods

### Literature search

Our analyses utilize MycoDB, a database of 4010 studies from 445 papers in which plants were inoculated with mycorrhizal fungi and plant growth was measured [57]. Our study focused on only AM fungi (2984 studies from 359 papers), and included any study that added inoculum, regardless of whether it was pure culture inoculum or not. To facilitate our exploration of local adaptation using this database, all studies were screened to identify whether the source location was given for at least two of the three components of interest: fungal inoculum, host plant, and soil. This screen yielded a total of 1170 individual studies from 139 published papers. Each study was classified as to whether plant and soil, fungus and soil, or fungus and plant were sympatric (i.e., derived from the same location) or allopatric (i.e., derived from different locations) (see Additional file 5: Methods S1, Additional file 6: Table S1).

### Dataset construction

From each study we extracted information on plant biomass with and without mycorrhizal inoculation. The question of an appropriate fitness proxy is a long-standing one, not only for plants but other species as well [see 61]. In general, fitness proxies are associated with survival, fecundity, or mating success. In plants, such proxies may include seed production, survival, or more commonly, plant size (for example, biomass, height) [62]. In mycorrhizal relationships, the main factors determining plant fitness can be classified as those enabling the organism to reach the reproductive state (“indirect” factors) and those contributing in a “direct” manner by influencing quality and quantity of the seeds themselves. Thus plant fitness in the context of mycorrhizal relationships can take the form of fecundity (seed number and quality) or indirect measures concerning the ability of the plant to regenerate or vegetative vigor (such as physiological state or the ability to acquire resources) [56]. Unfortunately, very few studies in our database report direct measures of plant fitness (such as survival or seed production) so we chose to utilize plant biomass, which is widely reported, as a proxy for plant fitness even though it is an indirect measure of plant fitness. While plants can benefit from mycorrhizal inoculation without increasing in biomass [63, 64], these studies are relatively rare and not sufficient in number for meta-analysis.

Our analysis consisted of studies that fit into two groups. The first set of studies, Analysis Group 1, examined the potential for local adaptation to alter the effect of mycorrhizal inoculation on host biomass between studies while the second set of studies, Analysis Group 2, directly tested for local or maladaptation by utilizing studies which manipulated sympatric and allopatric pairings in the same study. We constructed 13 explanatory variables (either categorical or continuous) for each study, 12 of which were previously used in Hoeksema et al. [23]. One variable, ORIGIN was unique to our analyses, and was used to test for local adaptation in between-studies analyses (Analysis Group 1). ORIGIN is a five-level categorical variable which allows us to explore how plant response to mycorrhizal inoculation is altered when individual components of the mutualism are sympatric or allopatric to one another. We defined allopatric combinations as originating from distances greater than 6 km from one another while sympatric combinations originated less than 1.5 km from one another (no combinations occurred between 1.5 km and 6 km apart). Levels of the ORIGIN variable are: ‘Sympatric’ (all components are from the same geographic location), ‘Allopatric’ (all components are from different geographic locations), ‘Fungi-Soil Sympatric’ (fungus and soil sympatric but allopatric to the plant), Plant-Fungi Sympatric (plant and fungus sympatric but allopatric to the soil), and Plant-Soil Sympatric (plant and soil sympatric but allopatric to the fungus). We could not investigate the degree to which distance between plant, fungal, or soil origin was directly responsible for shaping the effect of mycorrhizal inoculation on plant biomass because we could not appropriately calculate distances for all allopatric combinations in which all three components of the symbiosis are from different geographic locations. Thus, this analyses was beyond the scope of our current analyses.

Although local adaptation was the focus of the analyses and results presented here, the additional explanatory variables (besides ORIGIN) were included in analyses to account for unexplained variation in effect sizes and to obtain the most accurate estimates of local adaptation effects. Nine of the explanatory variables (outlined in Table 1) were treated as fixed effects. The remaining three random effect variables were categorical variables: plant taxonomy (PLANT SPECIES), a unique variable for each observation (EXPERIMENTID), and a unique variable for each set of observations that share a non-inoculated control (CTRLSETID). Additionally, when only a single species of fungal inocula was used, we used a random effect variable to control for the effect of fungal genus (FUNGALGENERA).

For between study analyses (Analysis Group 1), four overlapping sets of data were initially created to analyze the potential effect of local adaptation between each pair of the three components of interest: ‘Plant-Fungal’ which contained all studies with information about both the plant origin and the fungus origin, ‘Fungal-Soil’ which contained all studies with information about both the fungus and soil origin, ‘Plant-Soil’ which contained all studies with information about both the plant and soil origin, and ‘Plant-Fungal-Soil’ which only contained studies with information about the plant, fungus, and soil origins. Figure 1 is an overview of the available studies (and papers) for each of these datasets. To accurately evaluate the full extent to which local adaptation among plants, fungi, and soils plays in altering plant response to mycorrhizal inoculation, our analysis focused on those studies in which information about the plant, fungus, and soil origin was known (‘Plant-Fungal-Soil’). This dataset was parsed into smaller subsets to examine studies that contained (1) all available studies, (2) single species inocula only and (3) laboratory studies only. See Additional file 1: Table S2 for a full outline of which explanatory variables were included for each analysis subset. All data was deposited into Dryad: http://dx.doi.org/10.5061/dryad.723m1.

### Calculation of effect sizes and within-study variances

Plant response to mycorrhizal inoculation was quantified from data on whole plant biomass when it was available; otherwise, response was quantified with shoot biomass only. Separate analyses were conducted using total plant biomass and shoot biomass only, and results were substantively the same, so we report results from a combined analysis of both metrics of plant biomass. For Analysis Group 1, the effect size of inoculation was quantified using a standardized, unitless measure of performance in inoculated treatments relative to non-inoculated controls [23], the log response ratio of inoculated to non-inoculated plant biomass: ln(*X*_*inoc*_*/X*_*ctl*_) where *X*_*inoc*_ is the mean biomass in an inoculated treatment and *X*_*ctl*_ is the mean biomass in a non-inoculated control. Positive values of this metric indicate beneficial effects of mycorrhizal inoculation on plant biomass and negative values indicate detrimental effects of mycorrhizal inoculation on plant biomass.

Because 78 % of studies failed to report measures of dispersion for treatment means (e.g., variances or standard errors) we approximated the within-study variance associated with each effect size based on levels of replication in inoculated and non-inoculated treatments. The within-study variance used in our weighted regressions was estimated as:

$$ {\sigma}^2=\frac{1}{n_{inoc}}+\frac{1}{n_{ctl}} $$

where *n*_*inoc*_ and *n*_*ctl*_ are the number of replicates in inoculated and non-inoculated treatments.

### Calculation of within-paper effect sizes

Analysis Group 2, which consisted of studies that compare sympatric and allopatric pairings within a single study, allowed us to directly test the effect of local adaptation for altering plant response to mycorrhizal inoculation. Specifically, we compiled a subset of papers that studied both sympatric and allopatric combinations of plants and soil, plants and fungi, or fungi and soil. For this subset, we calculated the log response ratio of sympatric to allopatric effect sizes:

$$ Within\  Paper\  Effect\  Size= \log \left(\frac{X_{inoc,\ sym}}{X_{ctl,\ sym}}\right)- \log \left(\frac{X_{inoc,\  allo}}{X_{ctl,\  allo}}\right) $$

where ‘sym’ indicates sympatric pairings and ‘allo’ indicates allopatric pairings. Thus, when this effect size is positive, the relationship between the two components of interest (fungus, soil, plant) indicates local adaptation, and when the metric is negative, the components of interest indicate maladaptation. The estimated variance for this metric was calculated by combining the variance components for sympatric and allopatric pairings, which were calculated using the same variance estimator given above for the between-studies effect size. Explanatory variables were then checked to see how many observations per level remained and the dataset was altered as follows. All studies in this analysis were conducted in laboratories with sterilized background soil. When assessing plant-fungal adaptation we could only consider 254 studies (from 7 papers) of AM fungi with the explanatory variables PLANT FUNCTIONAL GROUP, INOCULUM COMPLEXITY, MICROBIAL AMENDMENT (none vs. wash), and FERTILIZATION (yes vs. no). When assessing fungal-soil adaptation we could only consider 217 studies (from 5 papers) of AM fungi with the explanatory variables PLANT FUNCTIONAL GROUP, INOCULUM COMPLEXITY, MICROBIAL CONTROL, and FERTILIZATION. When assessing plant-soil adaptation we could only consider 28 studies (from 3 papers) of AM fungi inoculated with a single species of fungi with the explanatory variables PLANT FUNCTIONAL GROUP and FERTILIZATION (yes vs. no).

### Mixed multi-factor meta-analysis

All analyses were done with R statistical software, version 3.1.3 [65]. Meta-analyses were conducted using the *rma.mv* function from the *metafor* package [66] with maximum likelihood estimation of parameters. When moderators were significant, differences between levels were determined using planned contrasts and the *glht* function from the *multcomp* package [67]. Marginal means for significant moderators and the overall model estimate were calculated using the *predict* function of the *stats* package [65].

## Additional files

Additional file 1: Table S2. Summary of which candidate explanatory variables were included in the analysis for each data subset (PDF 178 kb) Additional file 2: Figure S1. Mean Plant Response to Mycorrhizal Inoculation (PDF 113 kb) Additional file 3: Figure S2. Weighted mean effect sizes ± standard error for within-paper analyses (PDF 170 kb) Additional file 4: Figure S3. Weighted mean effect sizes ± standard error as a function of inoculation complexity for plant-fungal-soil analyses (PDF 100 kb) Additional file 5: Methods S1. Creation and Update of MycoDB. (PDF 275 kb) Additional file 6: Table S1. Paper codes for origin determination. (PDF 114 kb)
