# Supplementary material for: Home-field advantage? evidence of local adaptation among plants, soil, and arbuscular mycorrhizal fungi through meta-analysis
Source: BMC Evol Biol. 2016 Jun 10;16:122. doi: 10.1186/s12862-016-0698-9 (PMC4902977; doi:10.1186/s12862-016-0698-9)
Supplement: Additional file 1: Table S2. — Summary of which candidate explanatory variables were included in the analysis for each data subset (PDF 178 kb) [file 12862_2016_698_MOESM1_ESM.pdf]

Table S2. Summary of the candidate predictor variables that were included in the analysis for each data subset. An 'X' indicates an inclusion of a candidate predictor variable in the analyses of a particular data subset. The variables STERILITY and MICROBIAL AMENDMENT were only used if all studies were laboratory experiments.

**Explanatory Variable**	**Fungi Only**	**Single Species inocula only**	**Lab Studies Only**	**Within-Paper: Plant-Fungal**	**Within-Paper: Fungal-Soil**	**Within-Paper: Plant-Soil**
Plant Functional Group	x	x	x	x	x	x
Inoculum Complexity	x	---	x	x	x	---
Sterility	---	---	x	---	---	---
Microbial Amendment	---	---	x	x	x	---
Experimental Set-Up	x	x	---	---	---	---
N Fertilization	x	x	x	x	x	x
P Fertilization	x	x	x	---	x	---
Origin	x	x	x	---	---	---
