# Supplementary material for: Home-field advantage? evidence of local adaptation among plants, soil, and arbuscular mycorrhizal fungi through meta-analysis
Source: BMC Evol Biol. 2016 Jun 10;16:122. doi: 10.1186/s12862-016-0698-9 (PMC4902977; doi:10.1186/s12862-016-0698-9)

Figure S1. Mean Plant Response to Mycorrhizal Inoculation. Regardless of whether plant, fungus, and/or soil originate in sympatry (same origin) or in allopatry (different origin), the mean effect size of mycorrhizal inoculum on host biomass was positive for arbuscular mycorrhizal (AM) inoculation. Values shown represent the ratio of weighted mean effect sizes ± standard error. The dotted line indicates no response, values above the line indicate positive local adaptation, and values below the line indicate maladaptation.

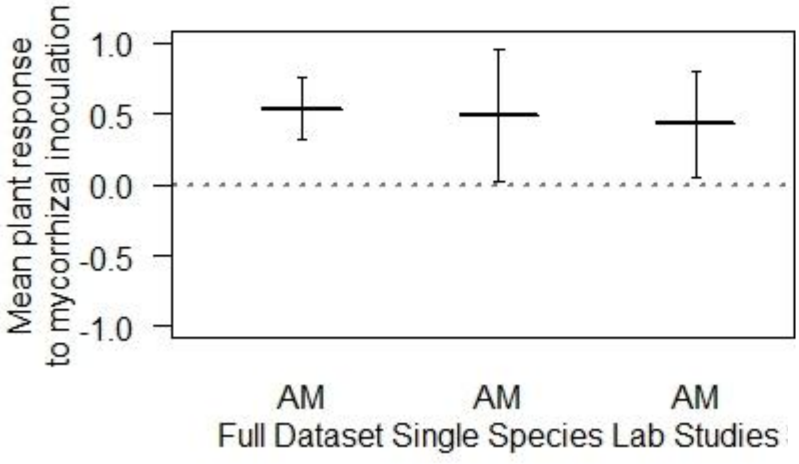

Supplement: Additional file 2: Figure S1. — Mean Plant Response to Mycorrhizal Inoculation (PDF 113 kb) [file 12862_2016_698_MOESM2_ESM.pdf]
