# Supplementary material for: Home-field advantage? evidence of local adaptation among plants, soil, and arbuscular mycorrhizal fungi through meta-analysis
Source: BMC Evol Biol. 2016 Jun 10;16:122. doi: 10.1186/s12862-016-0698-9 (PMC4902977; doi:10.1186/s12862-016-0698-9)

Figure S2. Inoculation Complexity for Plant-Fungal-Soil Analyses: When multiple species of fungi were used as inocula, the effect of mycorrhizal inoculation was greater than when the fungal inocula contained a single species or the whole soil. Values shown are weighted mean effect sizes ± standard error for arbuscular mycorrhiza from the Full Dataset (A) and Lab Studies (B). The dotted line indicates no response, values above the line indicate positive local adaptation, and values below the line indicate maladaptation. Symbols indicate differences from single species inocula based on planned contrasts. Significant codes: 0 '***' 0.001 '**' 0.01 '*' 0.05 '·' 0.1
.

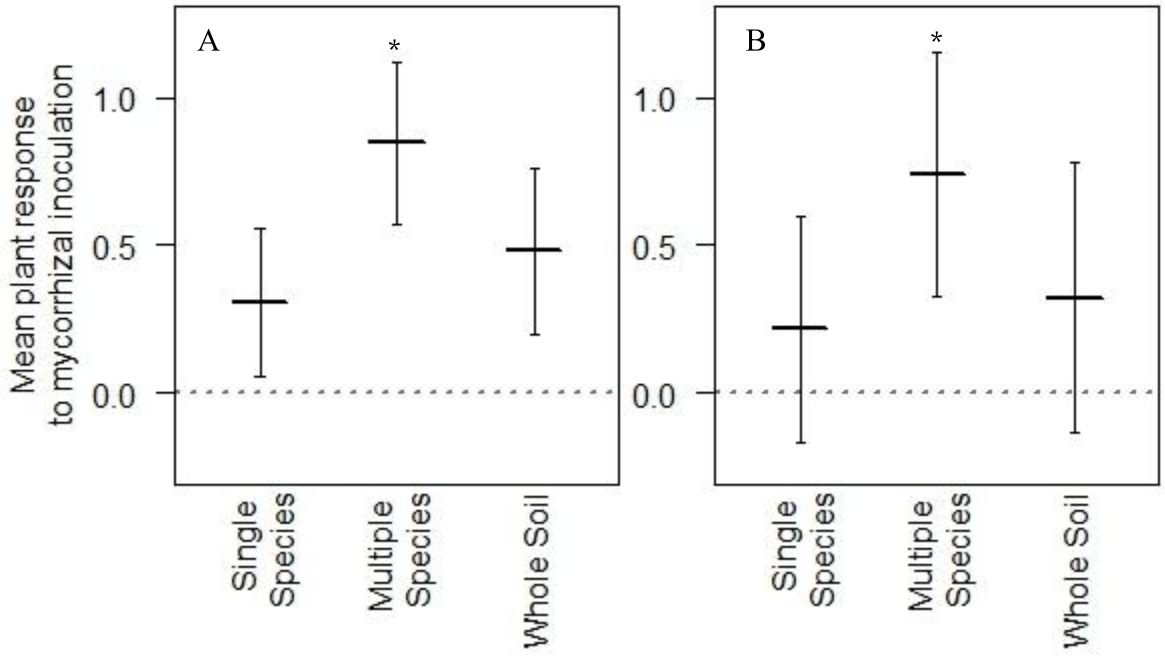

Supplement: Additional file 3: Figure S2. — Weighted mean effect sizes ± standard error for within-paper analyses (PDF 170 kb) [file 12862_2016_698_MOESM3_ESM.pdf]
