# Supplementary material for: Home-field advantage? evidence of local adaptation among plants, soil, and arbuscular mycorrhizal fungi through meta-analysis
Source: BMC Evol Biol. 2016 Jun 10;16:122. doi: 10.1186/s12862-016-0698-9 (PMC4902977; doi:10.1186/s12862-016-0698-9)

Figure S3. Mean Effect Size Ratio for Within Paper Analyses: None of the analyses have means that differed from zero, indicating no difference between biomass for allopatric pairings compaired to sympatric. Values shown represent the ratio of weighted mean effect sizes (ES) ± standard error for arbuscular mycorrhiza from within paper examinations of the plant and fungi, fungi and soil, and plant and soil. The dotted line indicates no response, values above the line indicate positive local adaptation, and values below the line indicate maladaptation.

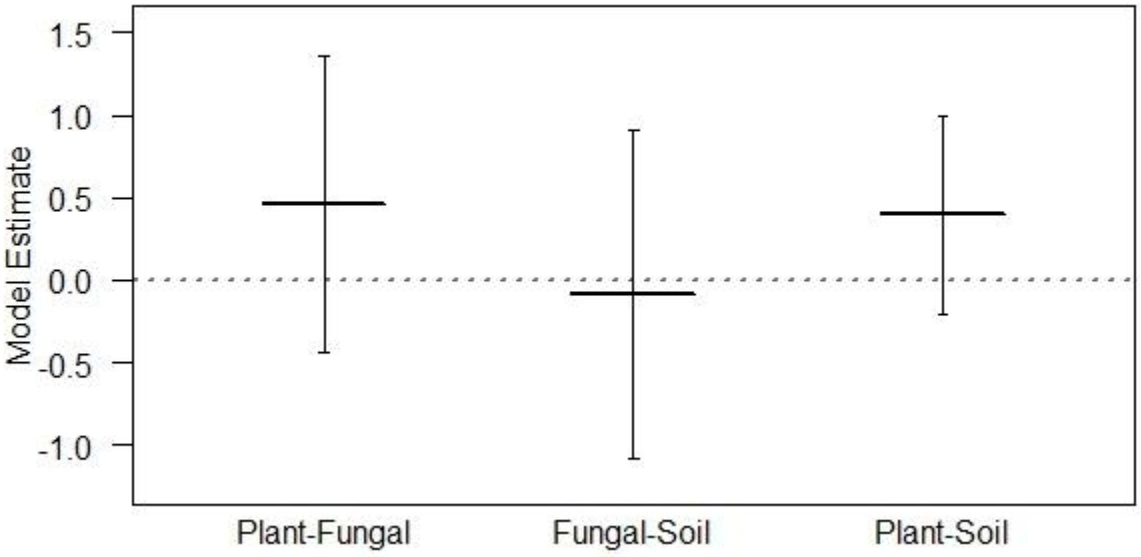

Supplement: Additional file 4: Figure S3. — Weighted mean effect sizes ± standard error as a function of inoculation complexity for plant-fungal-soil analyses (PDF 100 kb) [file 12862_2016_698_MOESM4_ESM.pdf]
