# Supplementary material for: Home-field advantage? evidence of local adaptation among plants, soil, and arbuscular mycorrhizal fungi through meta-analysis
Source: BMC Evol Biol. 2016 Jun 10;16:122. doi: 10.1186/s12862-016-0698-9 (PMC4902977; doi:10.1186/s12862-016-0698-9)
Supplement: Additional file 5: Methods S1. — Creation and Update of MycoDB. (PDF 275 kb) [file 12862_2016_698_MOESM5_ESM.pdf]

Methods S1. Creation and Update of MycoDB

On January 22, 2005 a list of 1852 peer-reviewed publications addressing the response of plants to mycorrhizal inoculation was identified from the ISI Web of Science database (1968-2004) by querying the database with the keywords *mycorrhiz** and *inoc** [see 1, 2]. A random subset of publications was screened for inclusion in previous analyses [1] and those publications were entered into a relational database called MycoDB designed to facilitate entry by multiple scientists and to allow for the production of customized outputs [3]. This initial random subset of studies contained substantially fewer studies involving EM fungi compared to AM fungi; therefore, an additional 49 publications which met our criteria and strictly focused on EM fungi were utilized from a previous analysis [4], which had obtained these studies from an ISI Web of Science search (1965-2006) using the keyword *ectomycorrhiza.*

As part of a Distributed Graduate Seminar (DGS) through the National Center for Ecological Analysis and Synthesis (NCEAS), an additional 1277 papers were added to the originial MycoDB [3]. Papers were found using multiple topic searches of ISI Web of Science on September 21, 2010. The "Overall" search used the key words: *(mycorrhiz** or *ectomyc** or *endomyc** or *arbuscul** or *vesicular**) and *inocul**. To enrich the data with respect to specific research questions, the following supplementary searches were conducted: A) Overall AND (*restoration* or *rehabilitation* or *reclamation* or *revegetation* or *reforestation*); B) Overall AND ('*local adaptation*' or *strain* or *isolate* or *genotype* or *ecotype* or *geograph**); C) Overall AND ('*tissueP*' or '*tissueN*' or '*shootP*' or '*shootN*' or '*leaf P*' or '*leaf N*'); D) Overall AND (*Gigaspor**or *Acaulospor** or *Scutellospora* or *Archaeospora*). The above searches resulted in a collection of 4013 papers. Articles that did not include both a fungal inoculation treatment and an uninoculated control were excluded.

As described in Hoeksema et al. [1], Chaudhary et al. [3], and Chaudhary et al. [2] individual papers could yield multiple studies for the purpose of our analysis. A 'study' was defined as a comparison of average plant performance between plants that were inoculated with mycorrhizal fungi and plants that were not inoculated. This criterion eliminated studies in which fungicide was added to plant roots to create different levels of colonization. In total, MycoDB now contains 4690 unique studies (from 1460 publications): 1167 studies (from 134 publications) as identified by Hoeksema et al. [1], 827 studies (from 49 publications) as identified by Karst et al. [4] and 2696 studies (from 1277 publications) as identified and entered by students of the NCEAS DGS [2].

References

1. Hoeksema JD, Chaudhary VB, Gehring CA, Johnson NC, Karst J, Koide RT, Pringle A, Zabinski C, Bever JD, Moore JC *et al*: **A meta-analysis of context-dependency in plant response to inoculation with mycorrhizal fungi**. *Ecology Letters* 2010, **13**(3):394–407.
2. Chaudhary VB, Antoninka A, Bever JD, Cannon J, Craig A, Gehring C, Ha M, Hart MM, Ji B, Johnson NC *et al*: **The context of mutualism: A global database of plant response to mycorrhizal fungi**. *Scientific Data In review*.
3. Chaudhary VB, Walters LL, Bever JD, Hoeksema JD, Wilson GWT: **Advancing Synthetic Ecology: A Database System to Facilitate Complex Ecological Meta-Analyses**. *Bulletin of the Ecological Society of America* 2010, **91**(2):235-243.
4. Karst J, Marczak L, Jones MD, Turkington R: **The mutualism-parasitism continuum in ectomycorrhizas: A quantitative assessment using meta-analysis**. *Ecology* 2008, **89**(4):1032-1042.
