# Supplementary material for: Home-field advantage? evidence of local adaptation among plants, soil, and arbuscular mycorrhizal fungi through meta-analysis
Source: BMC Evol Biol. 2016 Jun 10;16:122. doi: 10.1186/s12862-016-0698-9 (PMC4902977; doi:10.1186/s12862-016-0698-9)
Supplement: Additional file 6: Table S1. — Paper codes for origin determination. (PDF 114 kb) [file 12862_2016_698_MOESM6_ESM.pdf]

Table S1. Paper codes for origin determination where: X= unknown, Z= cultivar, 1=original location, 2 = location different from original, 3 = location different from both the original location and second location. Each study was assigned a code designating it as belonging to a specific combination of allopatric or sympatric combinations, differentiating between commercial potting soil/field soil and field collected/cultivated host plant. For example, if the source of both the host plant and fungi were known to be different, and they were grown in a potting soil mix, the study would be assigned a code of "I". If, however, the source of both the host plant and fungi were known to be different and they were grown in field soil from an unknown location, the study would be given a code of "G". Because we had large sample sizes without including the unknowns or cultivars, the results presented here do not consider unknowns or cultivars (i.e., we only used codes A-E).

Plant	Fungi	Soil	Code
1	1	1	A
1	1	2	B
1	2	1	C
2	1	1	D
1	2	3	E
1	1	X	F
1	2	X	G
1	1	Z	H
1	2	Z	I
1	X	1	J
1	X	2	K
X	1	1	L
X	1	2	M
Z	1	1	N
Z	1	2	O
